# Dental College Commencements

**Published:** 1889-05

**Authors:** 


### DENTAL COLLEGE COMMENCEMENTS.

### Ohio College of Dental Surgery.

The forty-third annual commencement of the Ohio College of Dental Surgery was held at College Hall, Cincinnati, Ohio, on Monday, March 4, 1889.

The number of matriculates for the session was one hundred and fifty.

The degree of D.D.S. was conferred on the following graduates by C. R. Taft, D.D.S., Vice-President of the Board of Trustees :

| NAME. | RESIDENCE. |
|---|---|
| Frank Archer Ayer | Ohio. |
| David William Barrow | Ohio. |
| Oscar L. Beard | Ohio. |
| Harry Clifton Blethrow | Pennsylvania. |
| Phares S. Bollinger | Ohio. |
| William T. Born | Ohio. |
| Ralph R. Braxtan | Indiana. |
| James T. Bristow | Michigan. |
| Asbury Ross Bryte | Pennsylvania. |
| Alva Matthews Bush | Ohio. |
| Clare Otis Carr | Ohio. |
| Frank L. Cary | Ohio. |
| Ben Carter Chandler | Kentucky. |
| William H. Cole. | Ohio. |
| Guss B. Cooper | Kentucky. |
| Alamander N. Copsey | California. |
| Charlie Henry Crigler | Kentucky. |
| Joseph M. Cunningham | New York. |
| William W. Dickey | Mississippi. |
| Felix William Doran | Iowa. |
| Benjamin F. Erb | Iowa. |
| George W. Gandee | Ohio |
| William Henry Gillett | Ohio. |
| Robert Lee Gray | Ohio. |
| Charles Henry Griffin | Ohio. |
| Melville McH. Haas | Indiana. |
| Horace Edwin Hatton | Ohio. |
| Robert H. Hodgen | Kentucky. |
| Aurdy Julian Holmes | Ohio. |
| Theophilus F. Hover | Pennsylvania. |
| William Oliver Hulick | Ohio. |
| Thomas O. Humphreys | Kentucky. |
| Harry Jenkins | Ohio. |
| Nathan Kelly | Illinois. |
| Edwin Franklin Keran | Minnesota. |
| Joshua R. King | Pennsylvania. |
| Will Clarence Lupfer | Ohio. |
| Herbert L. Madison | Iowa. |
| George Erwin Mann | Ohio. |
| Edwin Clark Maxwell | Michigan. |
| William T. McLean | Canada. |
| Mrs. Lucy D. Montz | Kentucky. |
| Edwin H. Moss | Missouri. |
| Henry Mathiot Patton | Ohio. |
| Fred. B. Pittwood | Illinois. |
| Eber Reynolds | Ohio. |
| Christopher C. Ritter | Indiana. |
| Carl Ritz | Ohio. |
| James E. Rothenbush | Ohio. |
| W. H. Sedgwick, jr | Ohio. |
| Horatio Clay Sexton | Indiana. |
| Edward G. Snodgrass | Ohio. |
| Elmer S. Snyder. | Iowa. |
| Jarome Walter Sparks | Indiana. |
| Andrew L. Sprague | Ohio. |
| Daniel H. Sullivan | Ohio. |
| Frank Berry Taylor | Kentucky. |
| Richard Taft Taylor | Ohio. |
| Horace M. Thomson | Kentucky. |
| Warren H. Upton | Iowa. |
| Lewis W. Watts | Ohio. |
| Arthur Jewell Weaver | Ohio. |
| Waitman A. Williams | Ohio. |
| Walter T. Withoft | Ohio. |
| George B. Wortman | Ohio. |

### Baltimore College of Dental Surgery.

The forty-ninth annual commencement of the Baltimore College of Dental Surgery was held at the Lyceum Theater, Baltimore, Md., on Friday, March 15, 1889, at 8 o'clock P.M.

The number of matriculates for the session was one hundred and eighteen.

The degree of D.D.S. was conferred on the following graduates by Professor R. B. Winder, Dean of the Faculty:

| NAME. | RESIDENCE. | NAME. | RESIDENCE. |
|---|---|---|---|
| G. E. Adams | New Jersey. | W. H. Lockwood | Wisconsin. |
| J. E. Armitage | New York. | J. H. Minard | Ontario. |
| L. P. Brown | New York. | F. H. Moore | Maine. |
| E. E. Butler | Virginia. | C. L. Morey | Texas. |
| P. C. Carmichael | New York. | E. E. Murray | North Carolina. |
| G. R. Carter. | Virginia. | J. F. McArthur. | Dakota. |
| G. E. Coughlin | Indiana. | T. S. McElfish | Maryland. |
| George B. Dorsey | Mississippi. | J. P. Nesbitt | Ohio. |
| A. R. Eaton | New Jersey. | O. M. Nisley | Indiana. |
| F. C. Exley | Georgia. | J. N. Penberthy | Minnesota. |
| N. L. Hale | Alabama. | E. G. Powers | Minnesota. |
| W. E. Hanah | Pennsylvania. | J. G. Robinson, Jr. | Maryland. |
| J. Hardy | Virginia | F. Rothenbach | Germany. |
| H. L. Harlan | Kentucky | G. B. Rush | Virginia. |
| L. F. Hough. | Virginia. | H. L. Sumption | Virginia. |
| G. W. S. Ireland | Maryland. | W. R. Spencer | Virginia. |
| W. D. James | Minnesota. | W. E. Walker | Mississippi. |
| B. B. Johnston | Dist. of Columbia. | J. R. Watson | Pennsylvania. |
| A. L. Jones | Virginia. | D. Williams | North Carolina. |
| W. C. Klatte | South Carolina. | J. P. Whedbee | Virginia. |
| A. Kraus | Roumania. | C. Whitney | Dist. of Columbia. |
| N. B. Larkin | Tennessee. | A. C. Whitehead | Virginia. |

### Indiana Dental College.

The tenth annual commencement exercises of the Indiana Dental College were held in the Young Men's Christian Association Hall, Indianapolis, Ind., on Wednesday, March 6, 1889, at 8 P. M.

The number of matriculates for the session was fifty-one.

The degree of D.D.S. was conferred on the following graduates by W. L. Heiskell, D.D.S., President of the Faculty:

| AME. | RESIDENCE. | NAME. | RESIDENCE. |
|---|---|---|---|
| M. F. Ault | Indiana. | Charles S. Hardy | Canada. |
| Peter S. Bower | Indiana. | Bion Moss | Wisconsin. |
| Ward E. Bullard | Indiana. | George B. Martin | Michigan. |
| Robert I. Blakeman | Kentucky. | Moses P. Niswouger | Ohio. |
| Harry W. Cole | Indiana. | Frederick H. Reiss | Illinois. |
| Waldo E. Callane | Indiana. | Charles K. Raber | Wisconsin. |
| Sidney W. Curtis | West Va. | W. Ellis Weissell | Indiana. |
| Willam Finn | Wisconsin. | John C. Walker | Kansas. |
| Willard W. Gates | Indiana. | | |

### Missouri Dental College.

The twenty-third annual commencement of the Missouri Dental College was held, in connection with that of the St. Louis Medical College, at Memorial Hall, St. Louis, Mo., on Thursday, March 14, 1889, at 7:30 P. M.

The number of matriculates for the session was fifty.

The degree of D.D.S. was conferred of the following graduater by Professor H. H. Mudd, M.D., Dean of the Faculty :

| NAME. | RESIDENCE. |
|---|---|
| Henry Allen Bragg | Missouri. |
| Jonathan Otis Eppright | Missouri. |
| John Thomas Fry | Missouri. |
| Charles Leroy Hickman | Missouri. |
| Philip Frank Hellmuth | Illinois. |
| William Worwick Holmes | Illinois. |
| Harry Taylor Hyams | Louisiana. |
| Gilbert, Wesley Jarvis | Missouri. |
| DeCourceyBradleyLindsley | Missouri. |
| Eugene Beauharinas Neal | Missouri. |
| Alexander Stark Oliver | Montana. |
| Geo. Harry Pipino, M.D | Illinois. |
| Julius Peter Ruge, M.D. | Missouri. |
| Henry Ruutz | Switzerland. |
| Edward A. F. Wulz | Missouri. |
| Frederick Edward Weber | Switzerland. |
| Joseph Henry Wilson | Missouri. |
| Adrien Zinsstag | Switzerland. |

The honorary degree of D.D.S. was conferred on Charles Rudolph Edward Koch, of Chicago, Ill.

### University of Maryland—Dental Department.

The seventh annual commencement of the Department of Dental Surgery of the University of Maryland was held at the Lyceum Theater, Baltimore, Md., on Wednesday, March 13, 1889.

The number of matriculates for the session was one hundred and twenty.

The degree of D.D.S. was conferred on the following graduates by Hon. S. Teackle Wallis, L.L.D., Provost of the University:

| NAME. | RESIDENCE. |
|---|---|
| Charles G. Aven | Virginia. |
| Augustine P. Badger | Maryland. |
| Eugene James Bailey | South Carolina. |
| Victor Durand Barbot | South Carolina. |
| Joseph Percy Blair | Virginia. |
| Frank Beck | Pennsylvania. |
| Kelly Ragland Bragg | Missouri. |
| H. Wood Campbell | Virginia. |
| T. S. D. Covington, Jr. | Virginia. |
| August A. T. W. Cuny | Germany. |
| E. Douglas Davis | W. Virginia. |
| Henry Davis, M.D | Missouri. |
| Edwin R. Dodson, M.D. | Maryland. |
| Pearl Louis Ellis | Vermont. |
| William L. Fish | New Jersey. |
| Harry Augustus Free | Pennsylvania. |
| David Goebricher | Maryland. |
| Joseph H. Haas | New York. |
| E. Patterson Hayes | Pennsylvania. |
| Joseph G. Heuisler | Maryland. |
| Reuben B. Hills | Massachusetts. |
| William H. Holland | South Carolina. |
| John W. McKinnon | Pennsylvania. |
| William Lee Miller | W. Virginia. |
| J. England Malony | South Carolina. |
| S. L. Nigolosian | Asia Minor. |
| Frank M. Oldham | South Carolina. |
| Czeslaus Opielinski | Germany. |
| Geo. B. Patterson | North Carolina. |
| Frank Zea Pirkey | California. |
| Benson S. Roberts | Bermuda. |
| F. F. W. Schloendorn | Germany. |
| Joseph B. Sharp | New Jersey. |
| Archie C. Shoemaker | Pennsylvania. |
| Benjamin Simons | South Carolina. |
| Hampton K. Smith | South Carolina. |
| C. vander Hoeven, M.D | Holland. |
| William J. Warnock | South Carolina. |
| M. V. Wright, M.D. | New Hampshire. |

### Columbian University—Dental Department.

The second annual commencement of the Dental Department of the Columbian University was held at Albaugh's Opera House, Washington, D. C., March 21, 1889.

The number of matriculates for the session was fourteen.

The degree of D.D.S. was conferred on the following graduates:

| NAME. | RESIDENCE. |
|---|---|
| John K. Halley | Dist. of Columbia. |
| Edith Jewell | Virginia. |
| Charces B. Munson | Virginia. |

### St. Louis College of Physicians and Surgeons—Dental Department.

The annual commencement exercises of the Dental Department of the St. Louis College of Physicians and Surgeons were held at Memorial Hall, St. Louis, Mo., on Friday, March 8, 1889.

The number of matriculates for the session was fourteen.

The degree of D.D.S. was conferred on the following graduates by Louis Bauer, M.D., Dean of the Faculty :

F. B. Chase,

T. F. Harrington,

Morris Israel,

George O. King,

Thomas M. Perrine,

Hanford Thayer.

### Southern Medical College—Dental Department.

The second annual commencement exercises of the Dental Department of the Southern Medical College were held at DeGiven's Opera House, Atlanta, Ga., on Saturday, March 2, 1889, an 8 o'clock P.M.

The number of matriculates for the session was thirty-six.

The degree of D.D.S. was conferred on the following graduates by T. S. Powell. M.D., President of the Board of Trustees ;

| NAME. | RESIDENCE. |
|---|---|
| J. A. Arbeely, M.D | Syria. |
| Aaron Branch | Georgia. |
| O. H. Cantrell | Georgia. |
| M. Z. Crist | Kentucky. |
| J. W. Daniel | Louisiana. |
| J. W. Duke | Georgia. |
| C. W. Forehand | Georgia. |
| H. J. Garland | Georgia. |
| S. M. Hyman | Georgia. |
| J. A. Link | Georgia. |
| S. M. Lide | Georgia. |
| R. L. Lane | Alabama. |
| B. R. McBath | Tennessee. |
| T. B. Pilcher | Georgia. |
| R. G. Regan | Alabama. |
| W. T. Sinclair | North Carolina. |
| H. B. Williamson | Alabama. |

### Kansas City Dental College.

The eighth annual commencement of the Kansas City Dental College was held, in connection with that of the Kansas City Medical College, at Music Hall, Kansas City, Mo., on Monday, March 11, 1889, at 8 o'clock P. M.

The number of matriculates for the session was thirty.

The degree of D.D.S. was conferred on the following graduates E. W. Schauffler, President of the Faculty :

| NAME. | RESIDENCE. | NAME. | RESIDENCE. |
|---|---|---|---|
| Rice R. Buchanan | Dakota. | Charles V. Larmar | Missouri. |
| Fritz Baum | Prussia. | Arthur E. McKellar | Kansas. |
| Ben. H. Dickson | Kansas. | Ross T. Thomas | Missouri. |
| Jefferson D. Hanna | Missouri. | Frynk L. Warren | Kansas. |
| Horace J. Hughs | Kansas. | Samuel C. Wheat | Missoure |
| Newton W. Hiatt | Indiana. | | |

### Howard University—Dental Department.

The annual commencement exercises of Howard University, including the Dental Department, were held at the Congregational Church, Washington, D. C., on Saturday evening, March 16, 1889.

The number of matriculates for the session was eleven.

The degree of D.D.S. was conferred on the following graduates by W. W. Patton, DD., L.L.D., President of the University:

| NAME. | RESIDENCE. | NAME. | RESIDENCE. |
|---|---|---|---|
| M. S. Campbell | Massachusetts. | Charles A. Neall | Massachusetts. |
| John J. Cary | Michigan. | Aug. C. Schwartz | Ohio. |
| J. M. Lamb, M. D. | Dist. of Columbia. | Hamilton Smith | Massachusetts. |

### Central Tennessee College—School of Dentistry.

The third annual commencement of the School of Dentistry of Meharry Medical Department of Central Tennessee College was held at Masonic Hall, Nashville, Tenn., February 21, 1889.

The degree of D.D.S. was conferred on the following graduates by J. Braden, D.D., President of the Faculty :

| NAME. | RESIDENCE. | NAME. | RESIDENCE. |
|---|---|---|---|
| Thos. Aulston Curtis | Alabama. | Jas. Bullock Maclin, M.D. | Louisiana. |
| Daniel Webster Fields | Tennessee. | Jas. Reynolds Porter, A.B. | Mississippi. |
| Stephen M. Hickman | Tennessee. | Alonzo Maury White | Tennessee. |

### Philadelphia Dental College.

The twenty-sixth annual commencement of the Philadelphia Dental College was held at the American Academy of Music, Philadelphia, on Thursday, February 28, 1889, at 8 P. M.

The number of matriculates for the session was two hundred and twenty-six. The degree of D.D.S. was conferred on the following graduates by the President of the Board of Trustees:

| NAME. | RESIDENCE. |
|---|---|
| Carlos Agnese | Brazil. |
| F. A. y Rodriguez, Ph.B. | Spain |
| Mary M. Apgar | Dist. Columbia. |
| Hester J. Baker | Illinois. |
| W. Egbert Barnes | Italy. |
| George W. Bates | Maine. |
| William W. Belcher | New York. |
| George R. Bell | Pennsylvania. |
| Percy Bennett | Australia. |
| Harry W. Bertholf | New Jersey. |
| Horace Betts | Delaware. |
| John H. Botz | Canada. |
| Peter C. Boulq | Alabama. |
| Camille Bourgeois | Louisiana. |
| Demosthenes Brush | New York. |
| Rubens S. Bustos | Chili. |
| Fred. J. Capon, L.D.S. | Canada. |
| George R. Churchill | Pennsylvania. |
| William A. Clymer | Minnesota. |
| Joseph P. Collins | Canada. |
| J. Charles Combe | Rhode Island. |
| F. Emanuel Doering | Canada. |
| Joseph E. Duffield | New Jersey. |
| George E. Ellerbeck | Utah. |
| Harold B. Findlay | Canada. |
| Andrew J. Flanagan | Massachusetts. |
| Annie T. Focht | Pennsylvania. |
| F. M. Fulkerson | Missouri. |
| Jesse P. Garvin | Wisconsin. |
| Fred W. Gibson | California. |
| John W. Gibson, M.D. | California. |
| Marinda B. Gifford | Massachusetts. |
| Thomas C. Gledhill | England. |
| Harry A. Glover | Indiana. |
| John W. Glover | Pennsylvania. |
| Arthur Y. Greene | Massachusetts. |
| A. DeWitt Gritman | New York. |
| Frederick L. Hamm | Ohio. |
| James N. Harris | Canada. |
| Frank A. Hatch | Massachusetts. |
| Charles Q. Hillegass | Pennsylvania. |
| William. M. Hodsdon | Massachusetts. |
| Joseph L. Golberg | Mississippi. |
| W. J. Hunt | Massachusetts. |
| Henry A. Ickes | Pennsylvania. |
| Aliee I. Ireland | New York. |
| J. William Jacob | Pennsylvania. |
| Benn C. Jewett | New York. |
| Albert B. Johnson | Connecticut. |
| Harvey C. Johnson | Pennsylvania. |
| G. Cleland Jones | Delaware. |
| James W. Joslyn | New Jersey. |
| Albert Kath | Germany. |
| Eugene L. Keen | Pennsylvania. |
| J. Edward Kelley | Ohio. |
| Arthur H. Kunze | Germany. |
| Anna K. Lettenmeier | Pennsylvania. |
| George R. Martin | Canada. |
| William R. Mills | New York. |
| Leslie D. Mitchell | New Jersey. |
| Richard D. McCaskey | Pennsyluania. |
| Herbert V. McCormick | New Jersey. |
| Edgar W. McFarland | Missouri. |
| A. Frederick Olmstead | New York. |
| John D. Powell | California. |
| Charles R. Pullen | Canada. |
| George I. Robb | Canada. |
| William S. Rosenthal | Pennsylvania. |
| D. B. Saxton. | Pennsylvania. |
| Richard Schmidt | Pennsylvania. |
| H. Hugo Schurig | Germany, |
| Cyrus U. Smith | Kansas. |
| S. C. Snyder | Illinois. |
| William A. Spring | Vermont. |
| Charles H. Stadlinger | New Jersey. |
| William S. Storer | New York. |
| Edward S. Talbot | Maine. |
| Charles B. Tiley | Connecticut. |
| Fred. R. Thompson | New York. |
| W. Irving Thompson | Kentucky. |
| Henry R. Thornton | Canada. |
| William F. Tremain. | New York. |
| Dalton Trumbauer | Pennsylvania. |
| T. T. Tunstall | Alabama. |
| Charles H. Turley | Pennsylvania. |
| Charles M. Vanderslice | Pennsylvania. |
| Charles W. Varcoe | New York. |
| David P. Ver Velen | Brazil. |
| A. T. Watson | Canada. |
| David H. Waugh | Canada. |
| B. Gifford Weber | Pennsylvania. |
| Harry W. Wellman | Missouri. |
| Albert C. White, Jr. | Rhode Island. |
| James G. Whiting | Illinois. |
| W. E. Willmott, L.D.S. | Canada. |
| J. Alexander Williams | Canada. |
| Dan. M. Wood | Indiana. |
| Eliza Yerkes | Pennsylvania. |

### University of Iowa—Dental Department.

The seventh annual commencement exercises of the Dental

Department of the State University of Iowa were held at the Opera House, Iowa City, Iowa, on Monday, March 4, 1889, at 8 o'clock P. M.

The number of matriculates for the session was seventy-nine.

The degree of D.D.S. was conferred on the following graduates by Charles A. Schaeffer, Ph.D., President of the University:

| NAME. | RESIDENCE. | NAME. | RESIDENCE. |
|---|---|---|---|
| Emma E. Auger | Iowa. | F. C. Noyes | Iowa. |
| George Burt Colt | Pennsylvania. | James A. Ogg | Minnesota. |
| E. Cotton | Iowa. | Edward Peck | Iowa. |
| Wm. Gilmore Clark | Iowa. | Albert D. Reed | Iowa. |
| Emma Eames Chase | Missouri. | Mrs. L. L. Richards, M.D. | Iowa. |
| George E, Diehl | Iowa. | Arthur T. Stillman | Iowa. |
| Kern M. Fullerton | Iowa. | Frank Slater | Iowa. |
| Arthur B. Glasier | Wisconsin. | G. Dayton Webb | Iowa. |
| William Humphrey | Iowa. | L. Alvin Young, M.D | Missouri. |
| Irving Burton Kenney | Iowa. | A. W. Zeigler | Wisconsin. |
| Ralph B. Murray | Dakota. | | |

### Royal College of Dental Surgeons of Ontario.

The examination at the Royal College of Dental Surgeons of Ontario was held in Toronto, March 5 to 8, inclusive, 1889. Excepting practical work the examination is wholly written.

The number of students in attendance for the session of 1888—89 was fifty-two.

The following obtained the diploma conferring the title of L.D.S., viz:

| NAME. | RESIDENCE. | NAME. | RESIDENCE. |
|---|---|---|---|
| N. W. Cleary | Renfew. | Charles S. McLean | Brockville. |
| E. Cunninghan | Collingwood. | G. P. Matthewman | Ottawa. |
| Edward Eidt | Berlin. | H. P. Martin | Toronto. |
| Charles Ferguson | London. | J. W. Oakley | Toronto. |
| A. Hugh Hipple | St. Catharines. | Andrew Rose | Picton. |
| J. T. Ireland | Seaforth. | C. A. Risk | Aberfeldy. |
| J. J. Kerr | Campbellford. | J. H. Swann | Toronto. |
| George McDonald | Arnprior. | A. J. Smith | Prescott. |
| R. G. McLaughlin | Brampton. | A. F. Webster, D.D.S | Toronto. |

### New York College of Dentistry.

The twenty-third annual commencement of the New York College of Dentistry was held at Chickering Hall, New York City, on Monday evening, March 11, 1889.

The number of matriculates for the session was two hundred and forty-five.

The degree of D.D.S. was conferred on the following graduates by William T. LaRoche, D.D.S., Vice-President of the Board of Trustees:

| NAME. | RESIDENCE. |
|---|---|
| Arthur Beaumont | England. |
| Wm. A. Berendsohn | New York. |
| Albert Bandmann | Germany. |
| Walter H. Bedell | New York. |
| Victor C. Bell | Russia. |
| H. N. Berthiaume | Rhode Island. |
| Frank W. Bridges | Brooklyn. |
| William N. Bush, Jr. | Brooklyn. |
| Edwin Cudlipp | New York. |
| Isaac W. Claypoole | New Jersey. |
| A. D'Orville Doty | New York. |
| John W. Davy | Ohio. |
| J. Marion Edmunds | Pennsylvania. |
| Stacy R. Everett | New Jersey. |
| Ralph W. Emerson | New Jersey. |
| Howard H. Fox | New York. |
| Enrique R. Gonzalez | Porto Rico. |
| Rudolph H. E. Gudewill | Germany. |
| James Galligher | New York. |
| Frank G. Gregory | Brooklyn. |
| Harry C. Green | Brooklyn. |
| Samuel Hess | New York. |
| Henry Heath, Jr | New York. |
| Robert G. Hutchinson, Jr | New York. |
| Cyrus A. Jordan, Jr | Long Island. |
| Wilhelm Kull | Switzerland. |
| Michael Leo | Germany. |
| Moritz H. Leukowicz | Austria. |
| E. F. Lauchantin | Brooklyn. |
| William S. Loomis | Iowa. |
| George MacNally | Argentine Repub. |
| Edward I. Mead | Connecticut. |
| Pablo R. Moreno | Mexico. |
| Ludwig M. Meyer | New York. |

| NAME. | RESIDENCE. |
|---|---|
| William H. Moon | Pennsylvania. |
| Michael Maskovich | Russia. |
| William S. May | Montana, |
| Norman S. Morgan | New York. |
| George E. McKugan | New Jersey. |
| Henry F. Maasch | Germany. |
| John B. Merritt | New York. |
| Ira L. Nickerson | New York. |
| John C. Oberle | New York. |
| Edward Owens | Ireland, |
| Henry P. Osborn | New Jersey. |
| Frank R. Parsons | Massachusetts. |
| George E. Pool | Brooklyn. |
| Edward H. Pease | Vermont. |
| A. W. Repelovski | New York. |
| Juan A. Riano | U. S. of Columbia. |
| William E. Rice | Connecticut. |
| Edwin S. Robinson | New York. |
| Alfred D. Seaver | New Jersey. |
| Abraham L. Sterne | Washington, D. C. |
| Max Sterne | Germany. |
| Frederick R. Smith | New York. |
| Abraham L. Smyth | New York. |
| Raphael Stork | Germany. |
| Primus C. Smith | Canada. |
| Clarence W. Steele | Vermont. |
| Edward H. Sears | Staten Island. |
| Edward H. Werner | Illinois. |
| Sigmund Wintner | Kentucky. |
| Robert M. Wollison | Massachusetts. |
| H. C. L. Weber | Finland. |
| William H. Webber | Massachusetts. |
| George E. T. Ward | Connecticut. |
| Charles S. Willson | Brooklyn. |
| Edward Waugh | Canada. |
| George E. Wilcox | Connecticut. |

### Vanderbilt University—Department of Dentistry.

The tenth annual commencement of the Department of Dentistry of Vanderbilt Uuiversity was held in the Masonic Theater, Nashville, Tenn., on Wednesday evening, February 20, 1889.

The number of matriculates for the session was ninety-five.

The degree of D.D. S. was conferred on the following graduates by Landon C. Garland, L.L.D., Chancellor of the University.

| NAME. | RESIDENCE. |
|---|---|
| J. W. Allen | Alabama. |
| T. C. Burgess | Tennessee. |
| C. S. Boyd | Alabama. |
| G. R. Crain | Tennessee. |
| J. M'Clark | |
| W. R. Desobry | Louisiana. |
| O. C. Farish | Alabama. |
| Jos. W. Ford | Kentucky. |
| J. C. Green | Alabama. |
| J. C. Goodwin | North Carolina. |
| W. C. Guess | Mississippi, |
| J. B. Hadden | Kentucky. |
| W. H. Helden | Alabama. |
| O. M. Huestis | Dakota. |

| NAME. | RESIDENCE. |
|---|---|
| R. P. Henderson | Alabama. |
| S. H. Johnson | Mississippi. |
| Robert Kettner | Michigan. |
| S. H. McKee | Georgia. |
| J. C. McDavitt | |
| A. A. McClannahan | Indiana. |
| O. McNeeley | Pennsylvania. |
| W. Norconk | Michigan. |
| D. F. Orr | Virginia. |
| J. L. Tichenor | Texas. |
| A. W. Trotter | Tennessee. |
| J. O. Templeton | Tennessee. |
| J. C. Wasson | Kentucky. |
| R. M. Walker | Illinois. |

### Pennsylvania College of Dental Surgery.

The thirty-third annual commencement of the Pennsylvania College of Dental Surgery was held at the American Academy of Music, Philadelphia, on Friday, March 1, 1889, at 8 P.M.

The number of matriculates for the session was one hundred and seventy-eight.

The degree of D.D.S. was conferred on the following graduates by S. W. Gross, M.D.; President of the Board of Trustees:

| NAME. | RESIDENCE. |
|---|---|
| Ambrose M. Allen | Pennsylvania. |
| Ismael Angulo | South America. |
| Clarence E. Bachman | Minnesota. |
| William C. Bailey | New York. |
| Alois Binotsch | Germany. |
| Scipio Bond | Minnesota. |
| C. Walter Borgner | Pennsylvania. |
| Frederick R. Brunet | Cuba. |
| Ramon Campuzano | Cuba. |
| Julia May Carmen | New York. |
| Geo. W. Chamberlain | New Jersey. |
| George M. Clark | New York. |
| Arthur Perry Clarke | New York. |
| Edw. Moberg Cooper | Pennsylvania. |
| W. M. G. Corrie, Ph. G. | Pennsylvania. |
| E. E. Culbert, L.D.S. | Canada. |
| E. C. Dean | Canada. |
| James L. Diven, Jr. | Pennsylvania. |
| J. Adams Dyer | Canada. |
| R. W. Edwards | New Jersey. |
| Leon A. Effron | Russia. |
| Milton R. Fisher | Louisiana. |
| Gertrude M. Flies | New York. |
| Lynn J. Gale | New York. |
| Clarke M. George | Ohio. |
| Frederick Tho. Gibson | Canada. |
| Albert H. Goodrich | Minnesota. |
| George N. Griswold | New York. |
| William E. Grover | Pennsylvania. |
| G. A. Guile | New York. |
| Wm. P. Gummer | California. |
| Zelopheard Hand | New Jersey. |
| Arthur H. Hanington | Canada. |
| Edgar Harding | Pennsylvania. |
| F. Kramer Heazelton | New Jersey. |
| Henry C. Heyer | Illinois. |
| Elmer Ellsworth Hill | Massachusetts. |
| Fannie E. Hoopes | Maryland, |
| James Homer Hope | Pennsylvania. |
| Fred. A. Hoyt | Minnesota. |
| W. Edwin Jackson | Pennsylvania. |
| E. M. Johnson | Minnesota. |
| O. Herschel Johnson | Pennsylvania. |
| Abner Jones | Ohio. |
| David Griffith Jones | New York. |
| Harry Davis Jones | Ohio. |
| N. H. Keyser | Pennsylvania. |
| Harvey C. King | Pennsylvania. |
| John R. Lane | Pennsylvania. |
| Chauncey G. Lewis | Ohio. |
| Elmer C. Lockard | New Jersey. |
| J. Dayton Lowrey | Pennsylvania. |
| Ernestine A. Mergler | Illinois. |
| John V. Mershon | Pennsylvania. |
| George Edwin Messick | Delaware. |
| W. M. Molyneaux | Pennsylvania. |
| Julio Moncada | South America. |
| Robert E. Morrison | Kentucky. |
| A. Fergus McBurney | Pennsylvania. |
| Thomas McCullough | Canada. |
| Kenneth McDougall | Pennsylvania. |
| Bert. V. Needham | New York. |
| Frank Culver Pague | California. |
| Wilber L Pepper | Pennsylvania. |
| Robert S. Ramsey | Canada. |
| John J. Reardon | New York. |
| Albin C. Rosenquist | Minnesota. |
| David A. Rosenthal | Russia. |
| Const'ino Eudoro Salcedo | South America. |
| Ignacio H. Santamaria | South America. |
| Fried Schlapp, M.C.D. | Germany. |
| C. Calvin G. Schomo | Pennsylvania. |
| Martha Schroeder | Germany. |
| John H. Shaw | Pennsylvania. |
| Matthew A. Smith | New Jersey. |
| William H. Snyder | Pennsylvania. |
| Richard S. Starkey | Canada. |
| F. Albert Stanger | New Jersey. |
| John C. Stites | Delaware. |
| William L. Straw | Pennsylvania. |
| T. C. Suganuma | Japan. |
| Wm. Rutherford Sutch | Pennsylvania. |
| Edward C. Truesdell | Minnesota. |
| Albert B. Van Osten | West Virginia. |
| M. L. Vansant, M.D. | Pennsylvania. |
| D. Porter Vincent | Pennsylvania. |
| Heinrich Volkmer | Germany. |
| W. W. Walker | Minnesota. |
| George C. Watson | Pennsylvania. |
| Irvin Norris Wells | Georgia. |
| Frank R. Zahniser | Pennsylvania. |

### University of Tennessee—Dental Department.

The eleventh annual commencement of the Dental Department of the University of Tennessee was held in the Masonic Theater, Nashville, Tenn., on Tuesday, February 26, 1889.

The number of matriculates for the session was twenty-two.

The degree of D.D.S. was conferred on the following graduates by Charles W. Dabney, Jr., Ph. D., President of the University:

| NAME. | RESIDENCE. |
|---|---|
| Charles H. Alexander | Tennessee. |
| Twiggs R. Boger | Georgia. |
| Arthur J. Cottrell | Tennessee. |
| Robert D. Crutcher | Tennessee. |
| Joseph B. Harris | Mississippi. |
| Eugene L. Holmes | Mississippi. |
| James H. Moore | Alabama. |
| Edward E. Slaton | Alabama. |
| Samuel J. Spargo | Tennessee. |
| J. C. Spivey | Mississippi. |
| W. Andrew Towns | Tennessee. |
| Alvin S. Willis | Tennessee. |
| W. M. Harris (Honorary) | Tennessee. |

